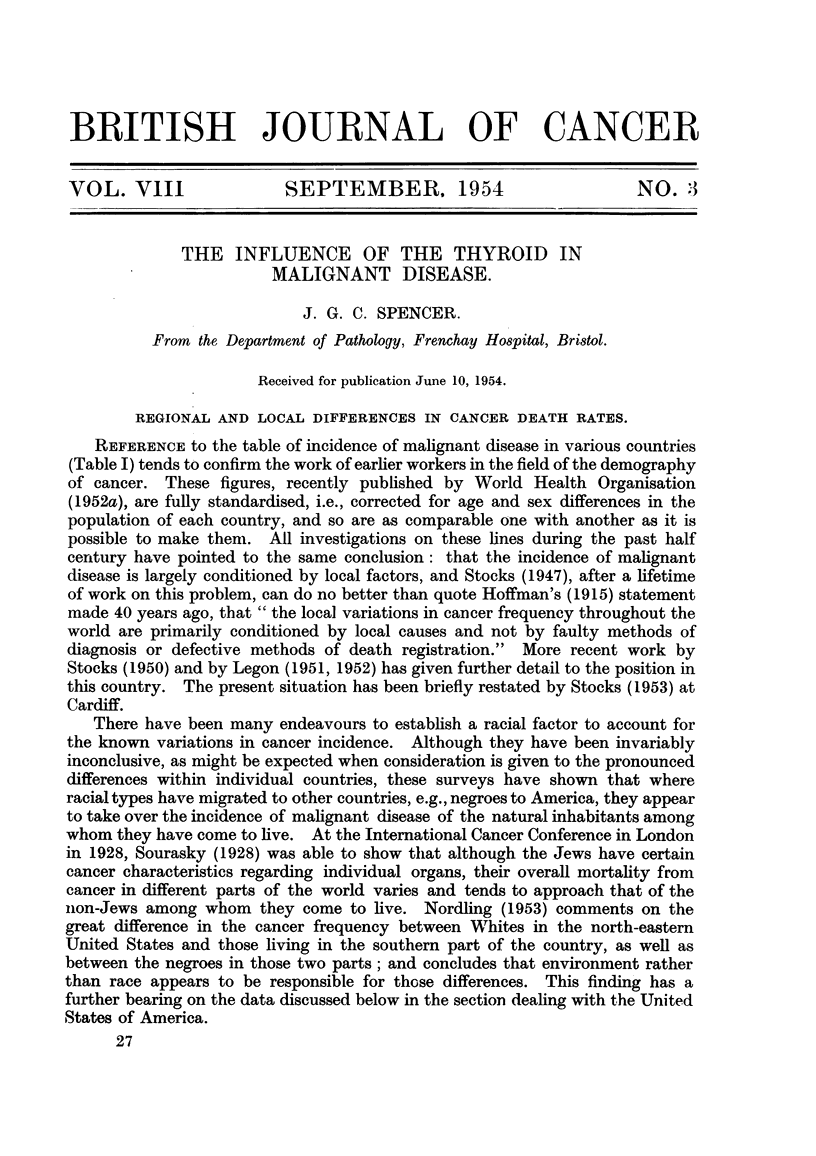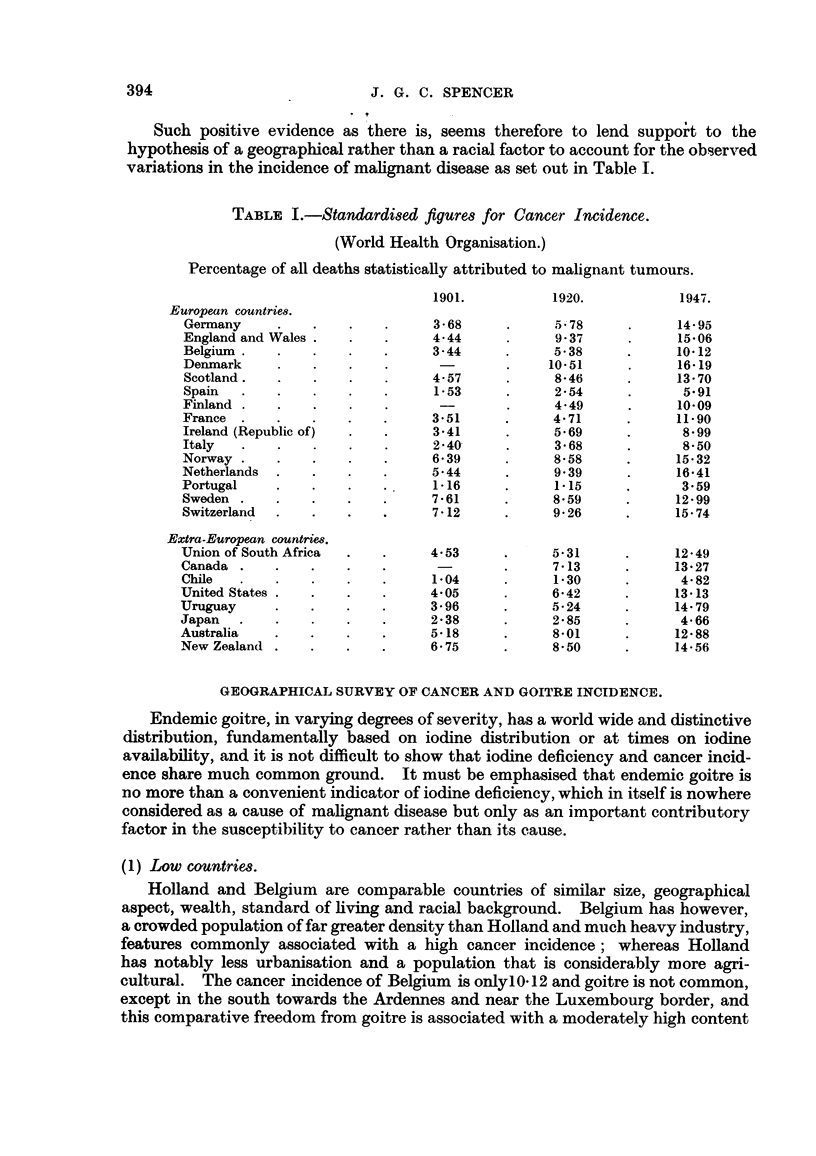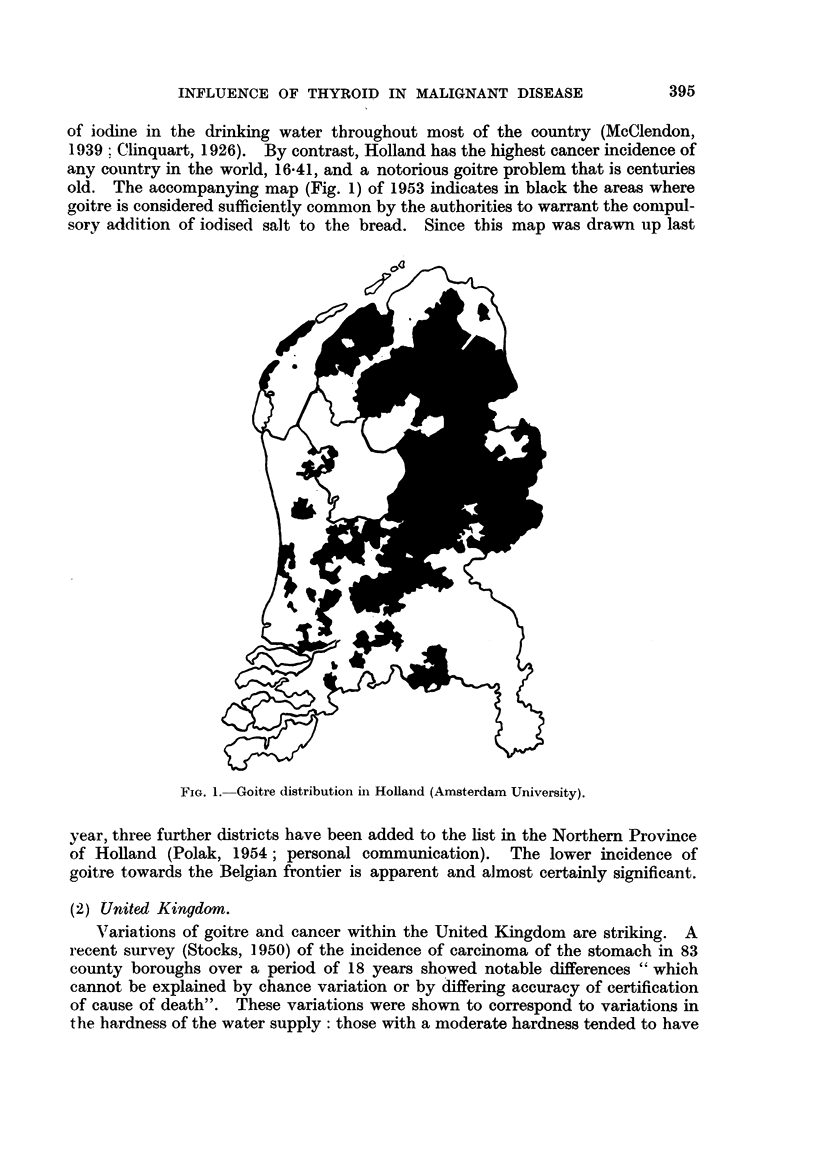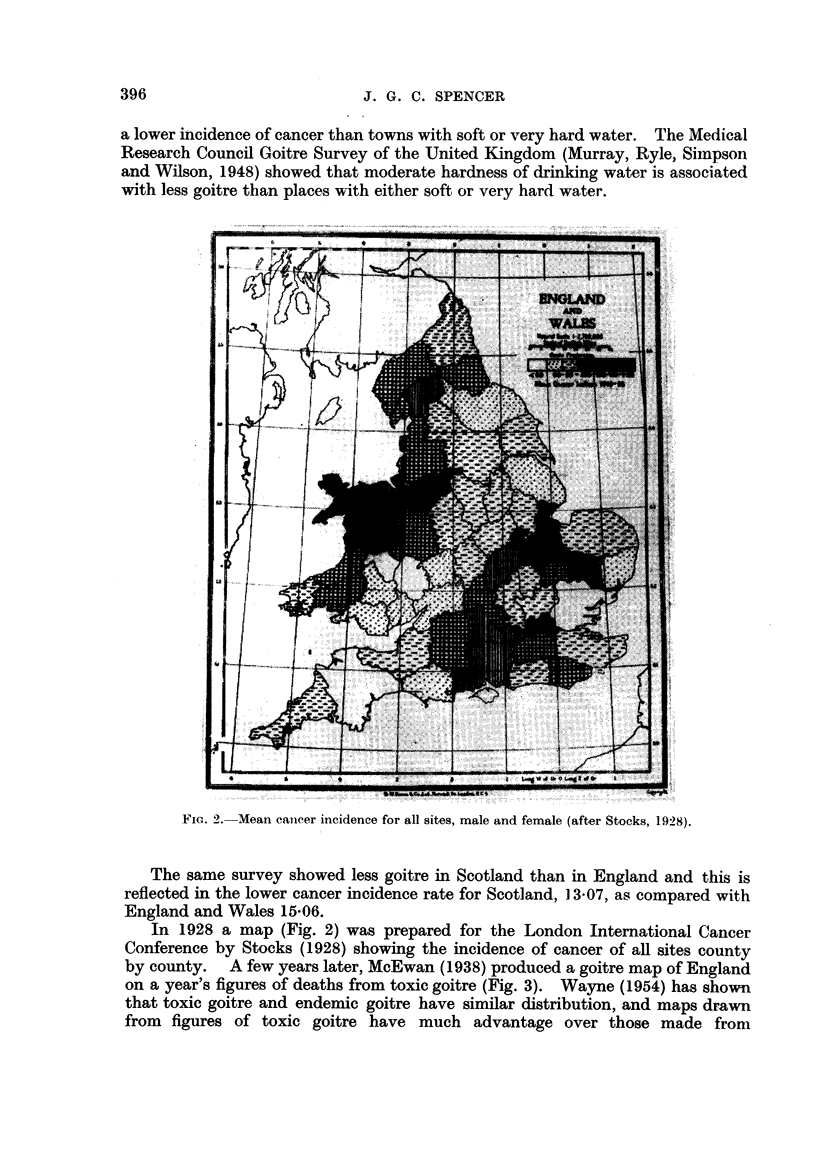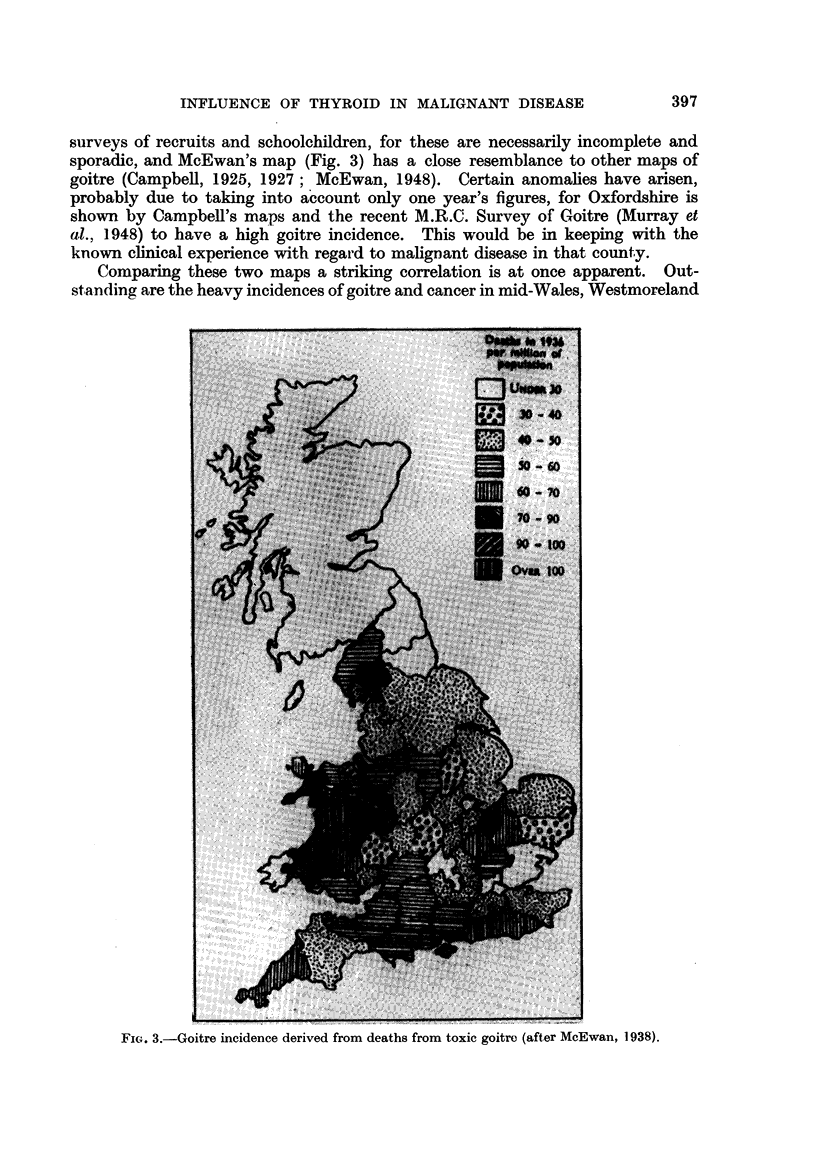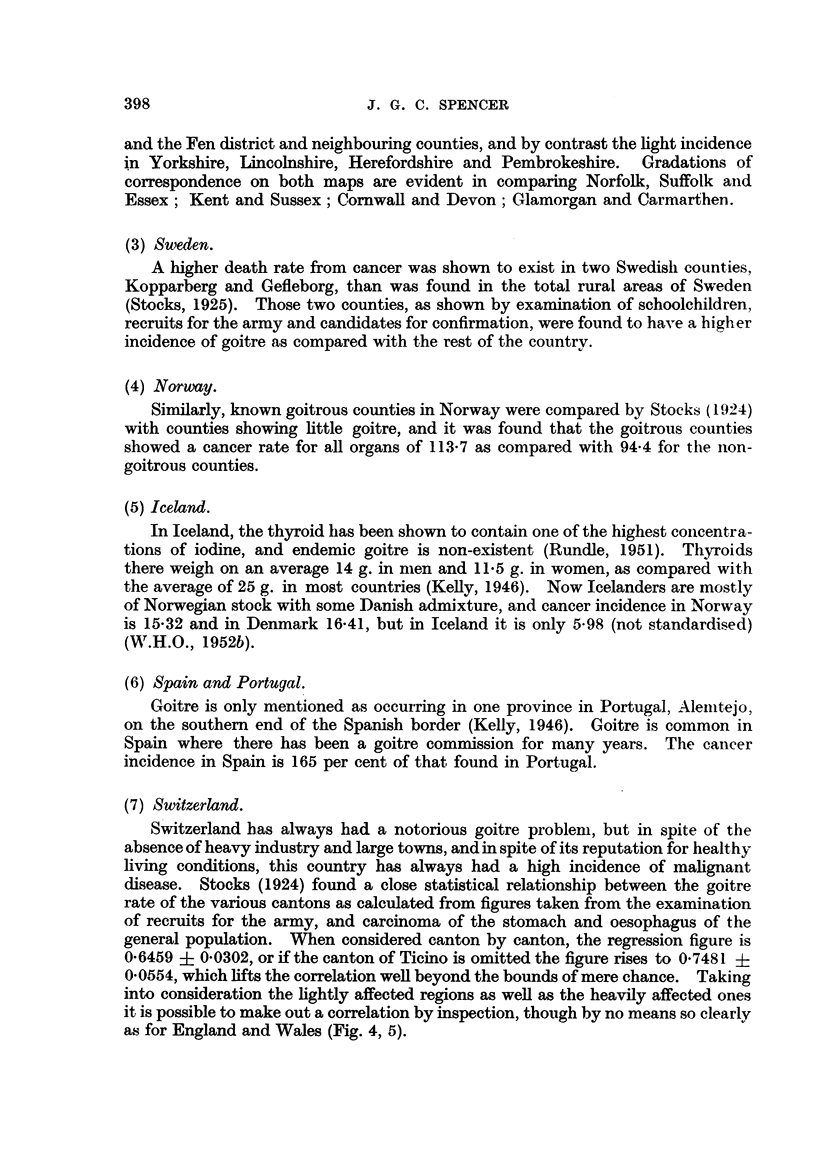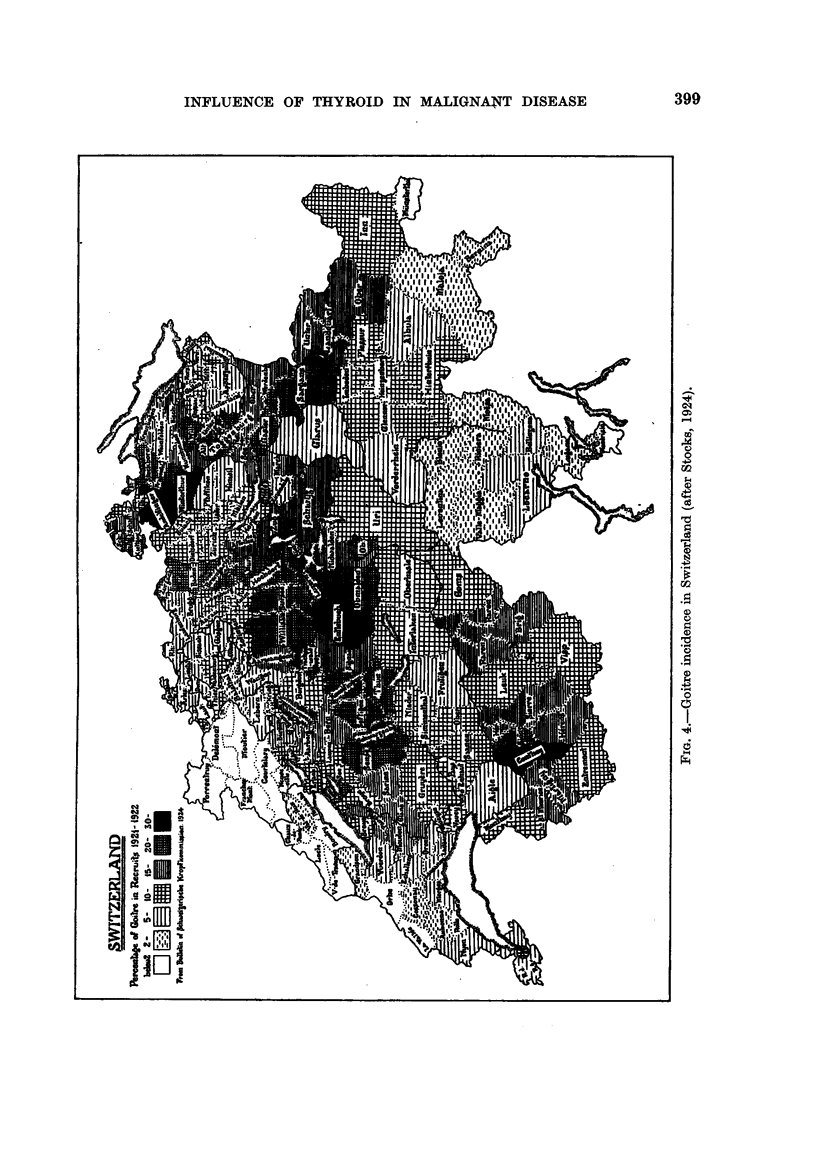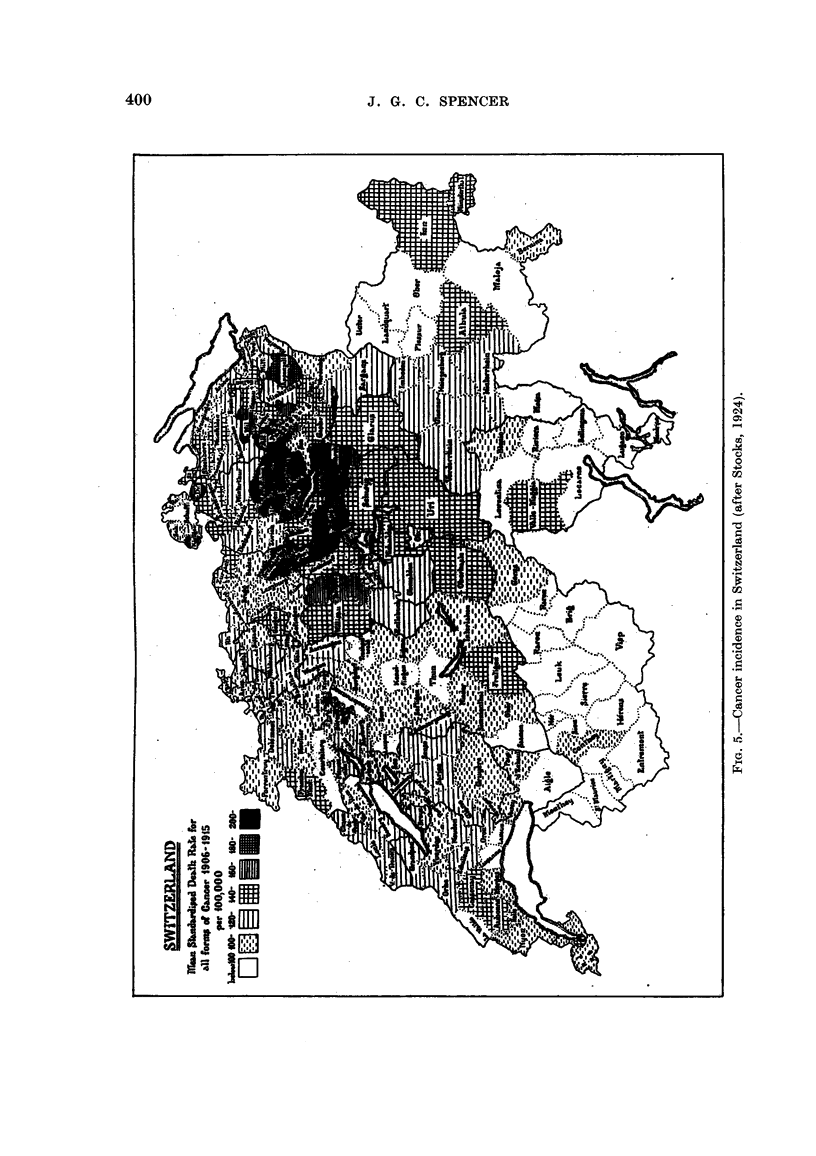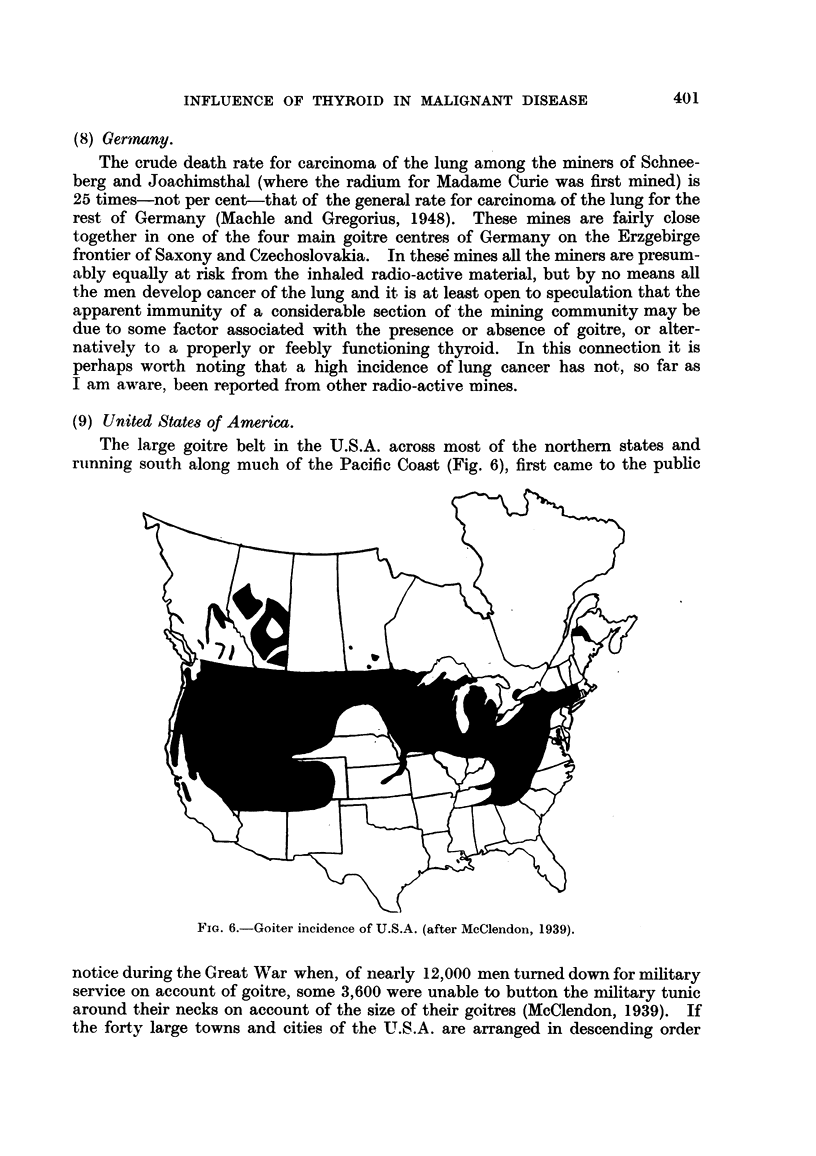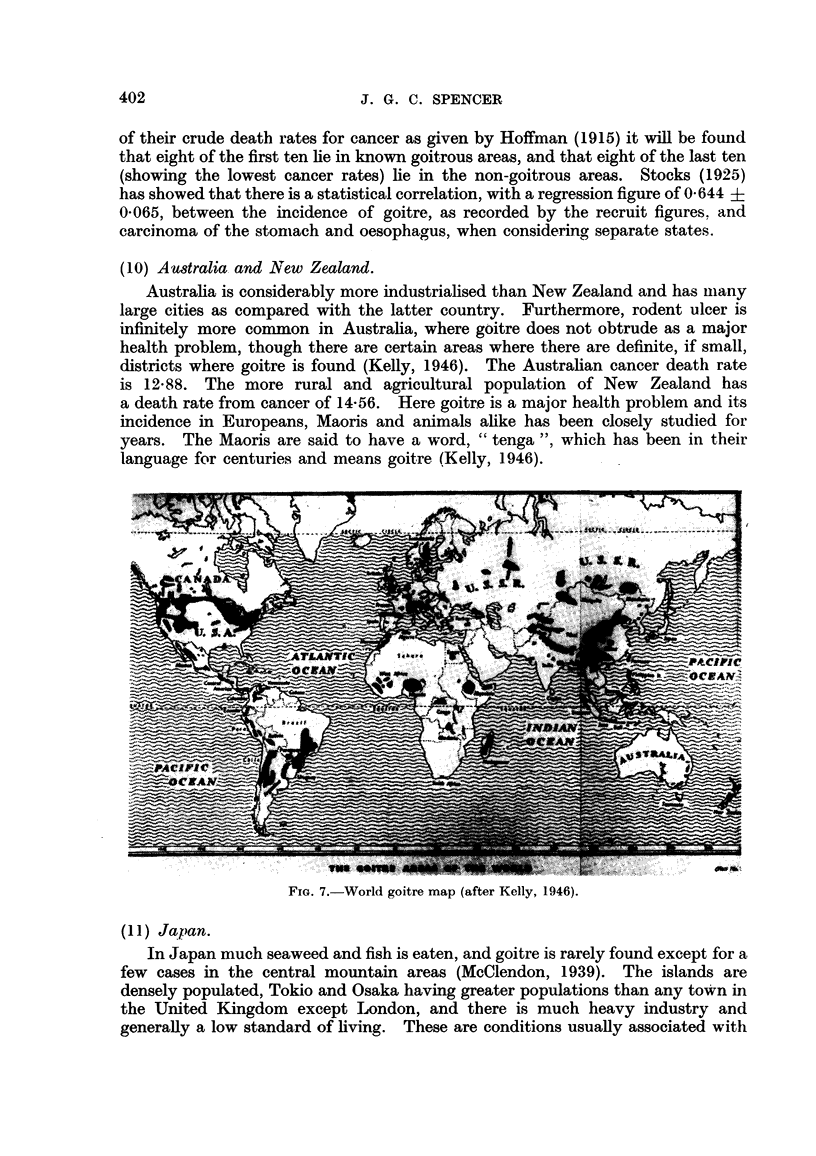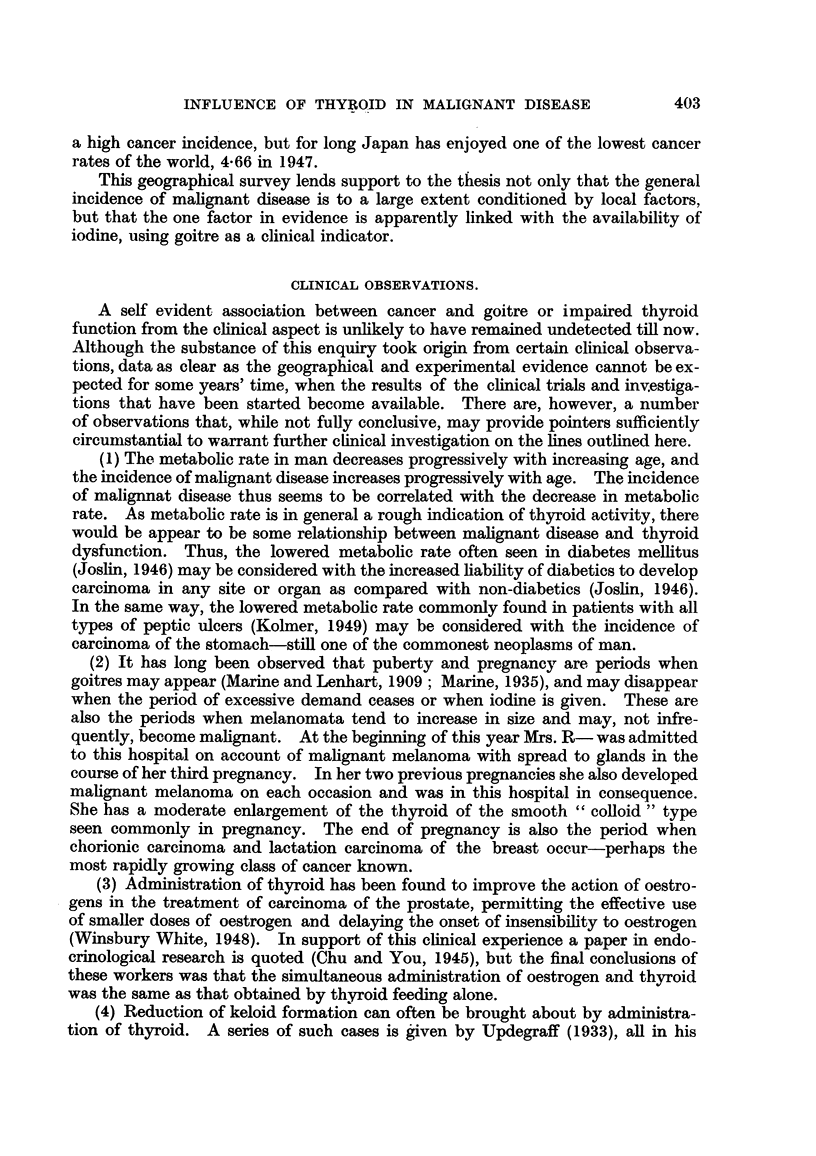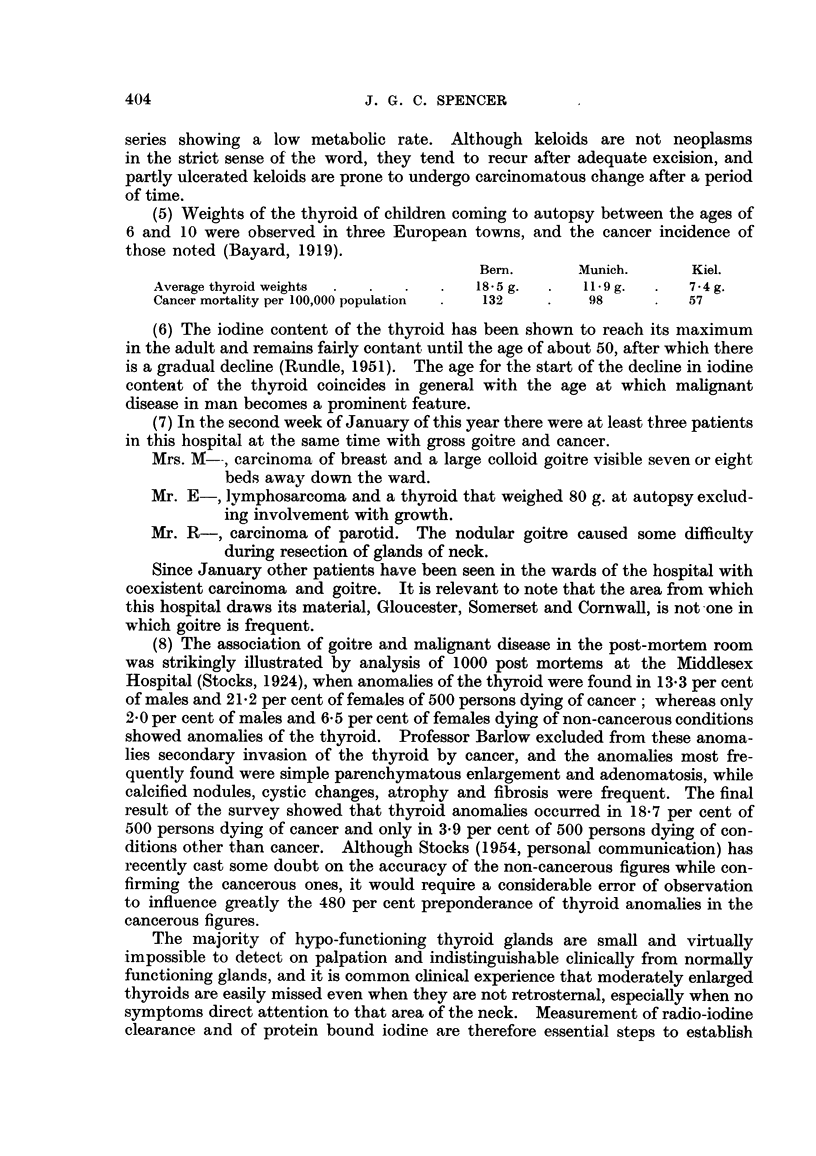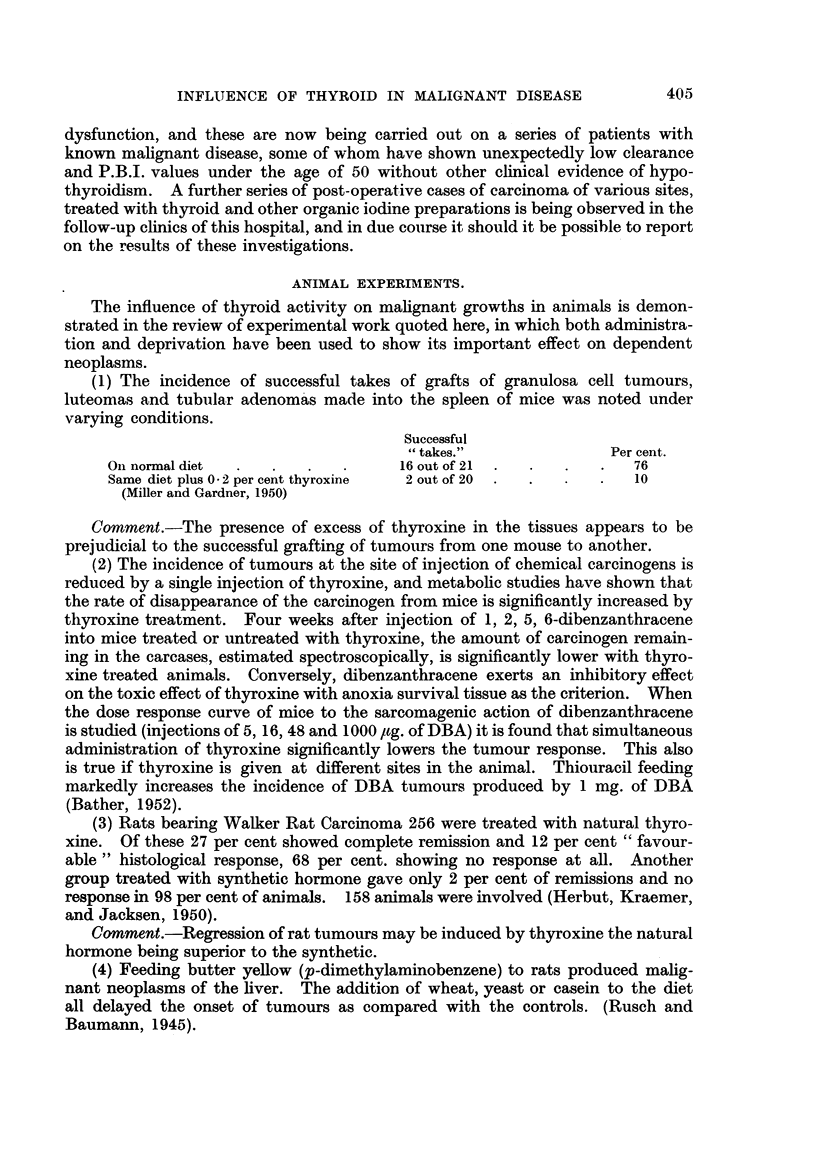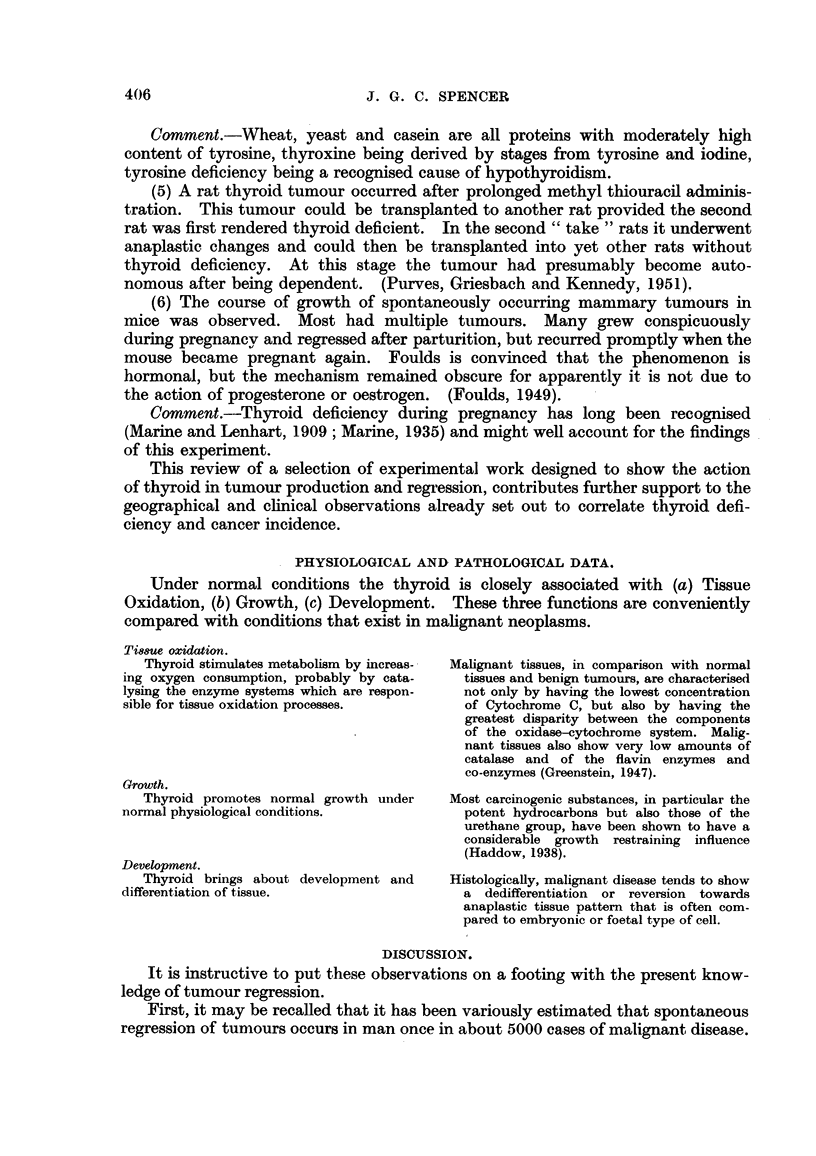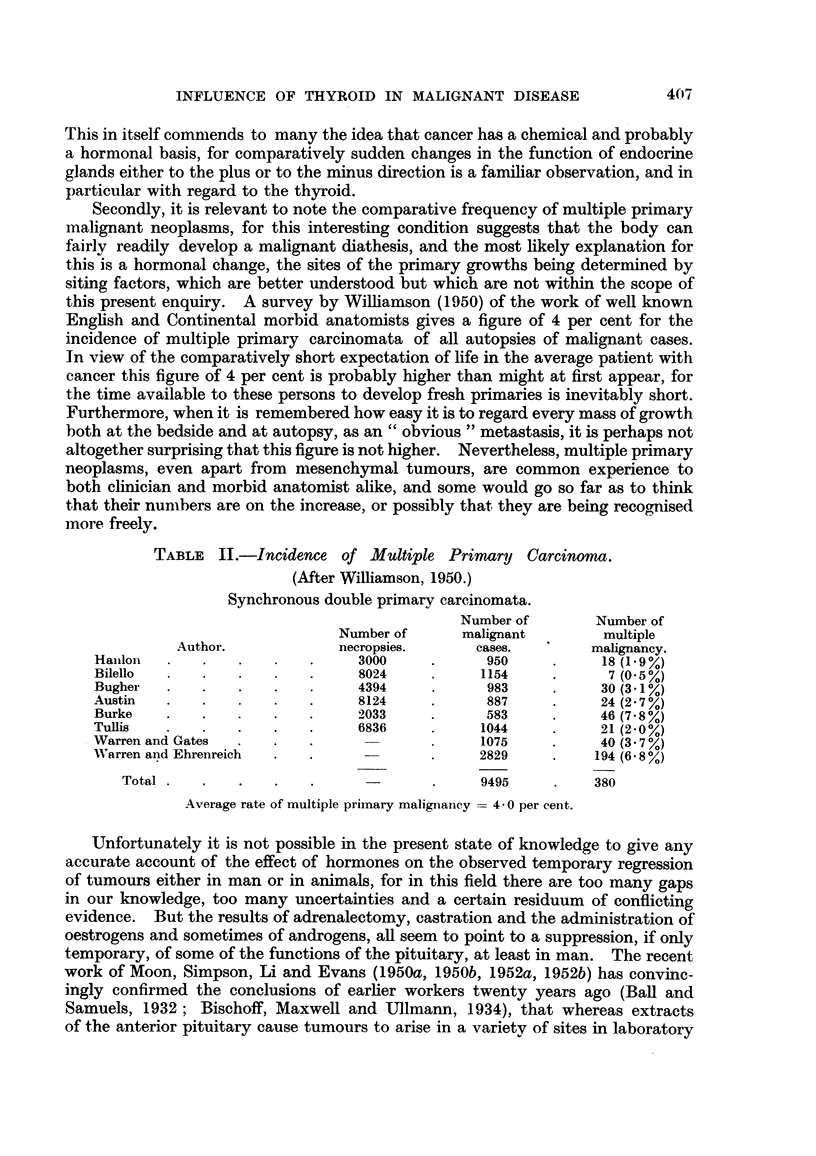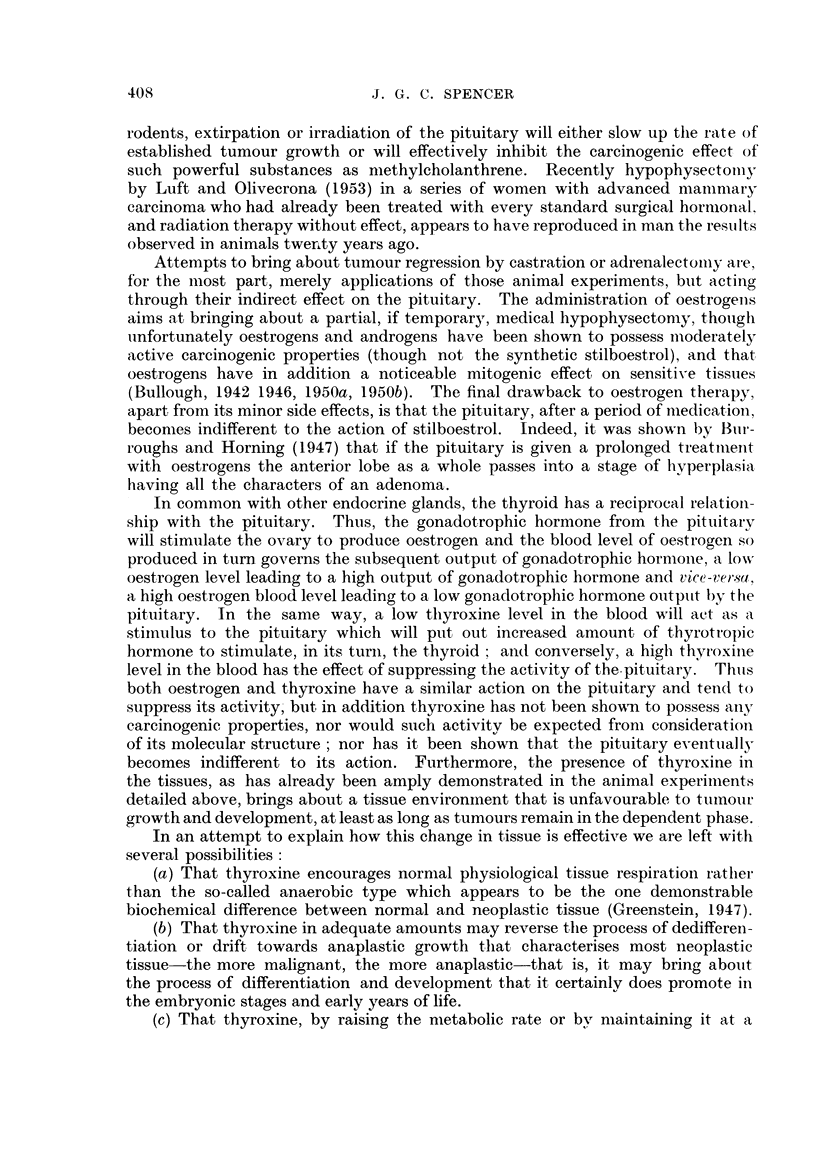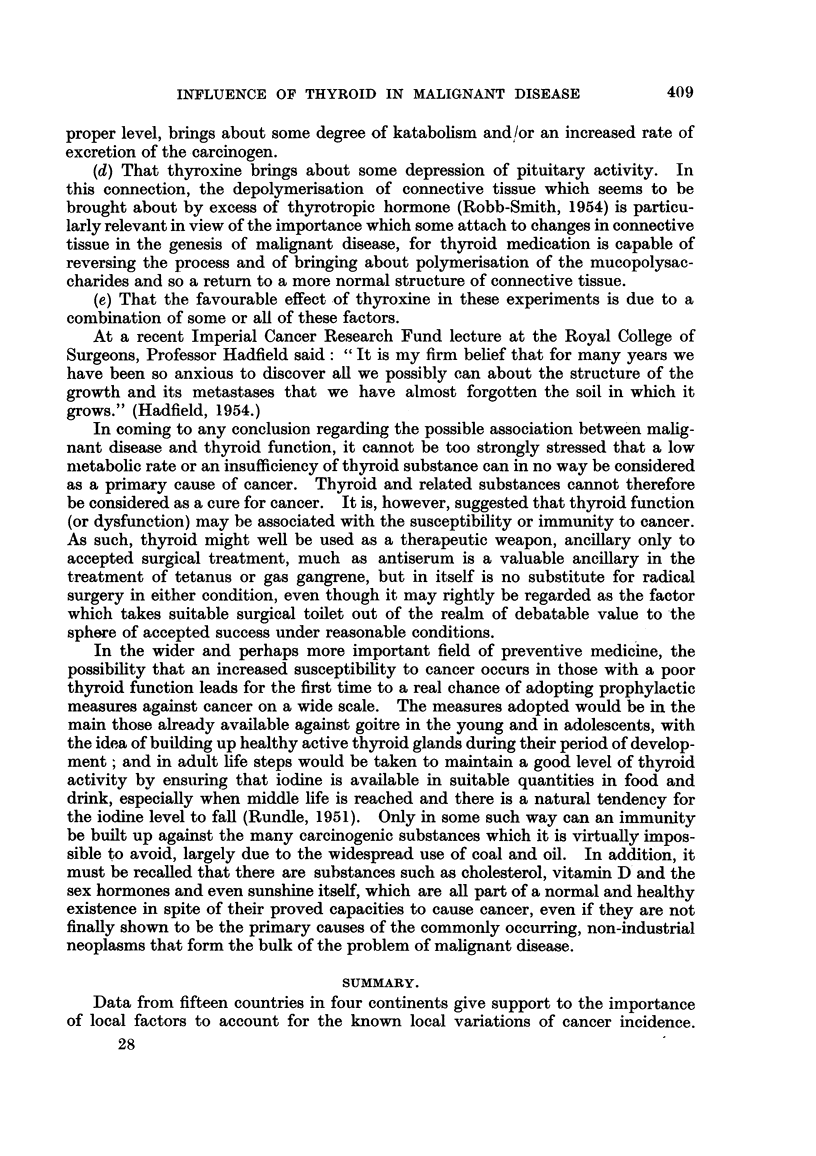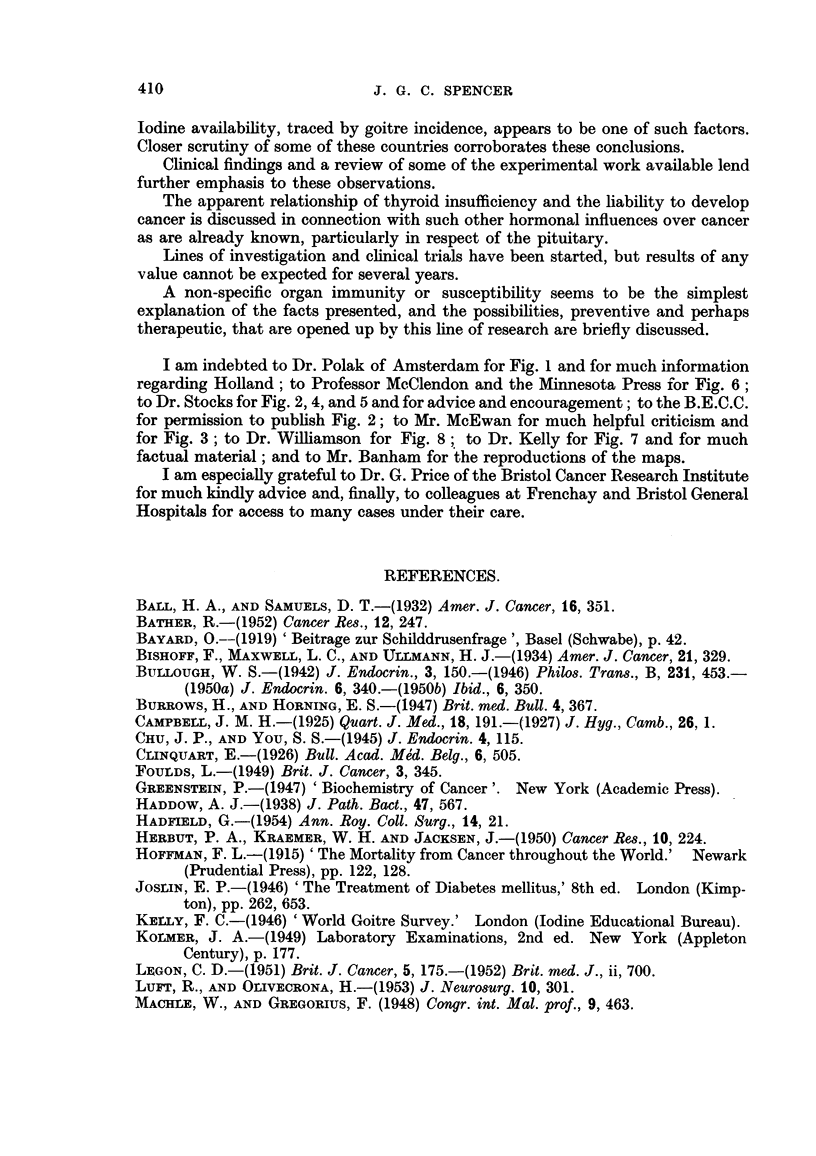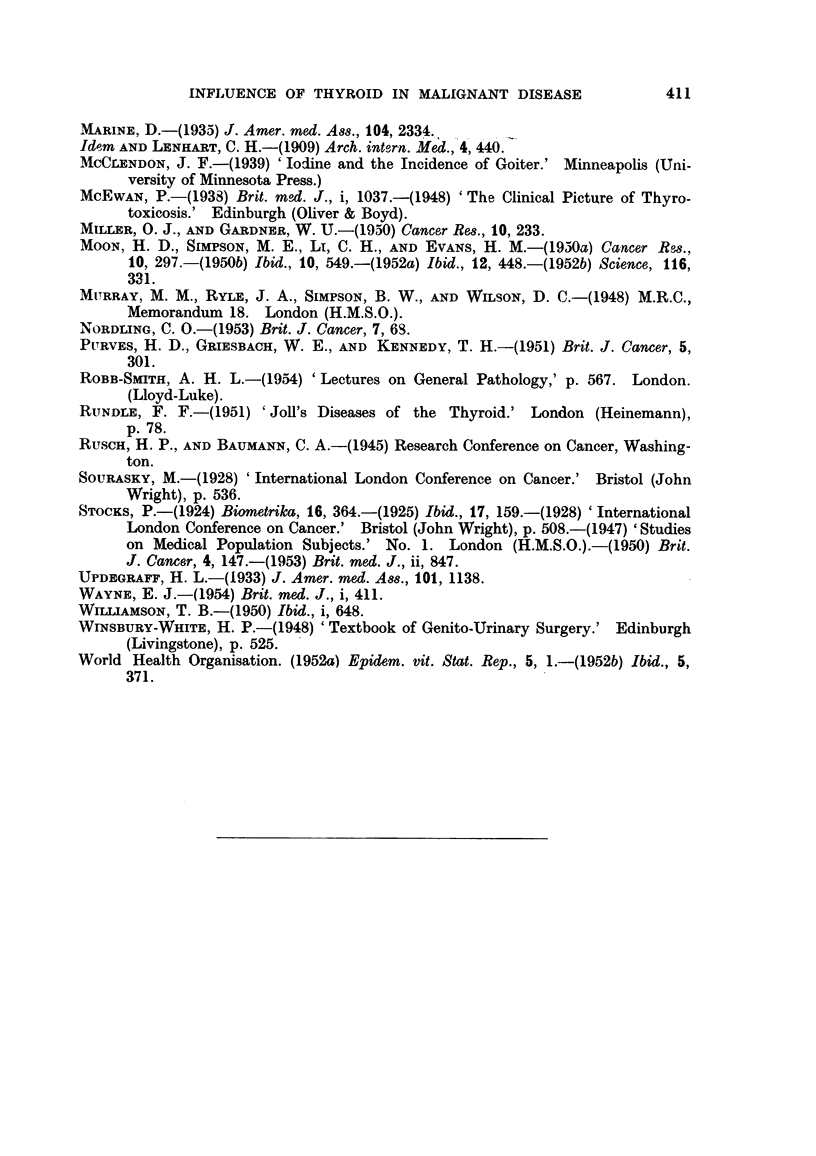# The Influence of the Thyroid in Malignant Disease

**DOI:** 10.1038/bjc.1954.41

**Published:** 1954-09

**Authors:** J. G. C. Spencer


BRITISH JOURNAL OF CANCER

VOL. VIII SEPTEMBER, 1954 NO. 3

### THE INFLUENCE OF THE THYROID IN MALIGNANT DISEASE.

J. G. C. SPENCER.

*From the Department of Pathology, Frenchay Hospital, Bristol.*

Received for publication June 10, 1954.

### REGIONAL AND LOCAL DIFFERENCES IN CANCER DEATH RATES.

REFERENCE to the table of incidence of malignant disease in various countries (Table I) tends to confirm the work of earlier workers in the field of the demography of cancer. These figures, recently published by World Health Organisation (1952*a*), are fully standardised, i.e., corrected for age and sex differences in the population of each country, and so are as comparable one with another as it is possible to make them. All investigations on these lines during the past half century have pointed to the same conclusion : that the incidence of malignant disease is largely conditioned by local factors, and Stocks (1947), after a lifetime of work on this problem, can do no better than quote Hoffman's (1915) statement made 40 years ago, that " the local variations in cancer frequency throughout the world are primarily conditioned by local causes and not by faulty methods of diagnosis or defective methods of death registration." More recent work by Stocks (1950) and by Legon (1951, 1952) has given further detail to the position in this country. The present situation has been briefly restated by Stocks (1953) at Cardiff.

There have been many endeavours to establish a racial factor to account for the known variations in cancer incidence. Although they have been invariably inconclusive, as might be expected when consideration is given to the pronounced differences within individual countries, these surveys have shown that where racial types have migrated to other countries, e.g., negroes to America, they appear to take over the incidence of malignant disease of the natural inhabitants among whom they have come to live. At the International Cancer Conference in London in 1928, Sourasky (1928) was able to show that although the Jews have certain cancer characteristics regarding individual organs, their overall mortality from cancer in different parts of the world varies and tends to approach that of the non-Jews among whom they come to live. Nordling (1953) comments on the great difference in the cancer frequency between Whites in the north-eastern United States and those living in the southern part of the country, as well as between the negroes in those two parts ; and concludes that environment rather than race appears to be responsible for those differences. This finding has a further bearing on the data discussed below in the section dealing with the United States of America.

Such positive evidence as there is, seems therefore to lend support to the hypothesis of a geographical rather than a racial factor to account for the observed variations in the incidence of malignant disease as set out in Table I.

TABLE I.—*Standardised figures for Cancer Incidence.*

(World Health Organisation.)

Percentage of all deaths statistically attributed to malignant tumours.

| | 1901. | 1920. | 1947. |
|---|---|---|---|
| *European countries.* | | | |
| Germany | 3·68 | 5·78 | 14·95 |
| England and Wales | 4·44 | 9·37 | 15·06 |
| Belgium | 3·44 | 5·38 | 10·12 |
| Denmark | — | 10·51 | 16·19 |
| Scotland | 4·57 | 8·46 | 13·70 |
| Spain | 1·53 | 2·54 | 5·91 |
| Finland | — | 4·49 | 10·09 |
| France | 3·51 | 4·71 | 11·90 |
| Ireland (Republic of) | 3·41 | 5·69 | 8·99 |
| Italy | 2·40 | 3·68 | 8·50 |
| Norway | 6·39 | 8·58 | 15·32 |
| Netherlands | 5·44 | 9·39 | 16·41 |
| Portugal | 1·16 | 1·15 | 3·59 |
| Sweden | 7·61 | 8·59 | 12·99 |
| Switzerland | 7·12 | 9·26 | 15·74 |
| *Extra-European countries.* | | | |
| Union of South Africa | 4·53 | 5·31 | 12·49 |
| Canada | — | 7·13 | 13·27 |
| Chile | 1·04 | 1·30 | 4·82 |
| United States | 4·05 | 6·42 | 13·13 |
| Uruguay | 3·96 | 5·24 | 14·79 |
| Japan | 2·38 | 2·85 | 4·66 |
| Australia | 5·18 | 8·01 | 12·88 |
| New Zealand | 6·75 | 8·50 | 14·56 |

### GEOGRAPHICAL SURVEY OF CANCER AND GOITRE INCIDENCE.

Endemic goitre, in varying degrees of severity, has a world wide and distinctive distribution, fundamentally based on iodine distribution or at times on iodine availability, and it is not difficult to show that iodine deficiency and cancer incidence share much common ground. It must be emphasised that endemic goitre is no more than a convenient indicator of iodine deficiency, which in itself is nowhere considered as a cause of malignant disease but only as an important contributory factor in the susceptibility to cancer rather than its cause.

(1) *Low countries.*

Holland and Belgium are comparable countries of similar size, geographical aspect, wealth, standard of living and racial background. Belgium has however, a crowded population of far greater density than Holland and much heavy industry, features commonly associated with a high cancer incidence; whereas Holland has notably less urbanisation and a population that is considerably more agricultural. The cancer incidence of Belgium is only10·12 and goitre is not common, except in the south towards the Ardennes and near the Luxembourg border, and this comparative freedom from goitre is associated with a moderately high content of iodine in the drinking water throughout most of the country (McClendon, 1939 ; Clinquart, 1926). By contrast, Holland has the highest cancer incidence of any country in the world, 16·41, and a notorious goitre problem that is centuries old. The accompanying map (Fig. 1) of 1953 indicates in black the areas where goitre is considered sufficiently common by the authorities to warrant the compulsory addition of iodised salt to the bread. Since this map was drawn up last

FIG. 1.—Goitre distribution in Holland (Amsterdam University).

year, three further districts have been added to the list in the Northern Province of Holland (Polak, 1954 ; personal communication). The lower incidence of goitre towards the Belgian frontier is apparent and almost certainly significant.

(2) *United Kingdom.*

Variations of goitre and cancer within the United Kingdom are striking. A recent survey (Stocks, 1950) of the incidence of carcinoma of the stomach in 83 county boroughs over a period of 18 years showed notable differences " which cannot be explained by chance variation or by differing accuracy of certification of cause of death". These variations were shown to correspond to variations in the hardness of the water supply : those with a moderate hardness tended to have a lower incidence of cancer than towns with soft or very hard water. The Medical Research Council Goitre Survey of the United Kingdom (Murray, Ryle, Simpson and Wilson, 1948) showed that moderate hardness of drinking water is associated with less goitre than places with either soft or very hard water.

FIG. 2.—Mean cancer incidence for all sites, male and female (after Stocks, 1928).

The same survey showed less goitre in Scotland than in England and this is reflected in the lower cancer incidence rate for Scotland, 13·07, as compared with England and Wales 15·06.

In 1928 a map (Fig. 2) was prepared for the London International Cancer Conference by Stocks (1928) showing the incidence of cancer of all sites county by county. A few years later, McEwan (1938) produced a goitre map of England on a year's figures of deaths from toxic goitre (Fig. 3). Wayne (1954) has shown that toxic goitre and endemic goitre have similar distribution, and maps drawn from figures of toxic goitre have much advantage over those made from surveys of recruits and schoolchildren, for these are necessarily incomplete and sporadic, and McEwan's map (Fig. 3) has a close resemblance to other maps of goitre (Campbell, 1925, 1927 ; McEwan, 1948). Certain anomalies have arisen, probably due to taking into account only one year's figures, for Oxfordshire is shown by Campbell's maps and the recent M.R.C. Survey of Goitre (Murray *et al.*, 1948) to have a high goitre incidence. This would be in keeping with the known clinical experience with regard to malignant disease in that county.

Comparing these two maps a striking correlation is at once apparent. Outstanding are the heavy incidences of goitre and cancer in mid-Wales, Westmoreland

FIG. 3.—Goitre incidence derived from deaths from toxic goitre (after McEwan, 1938).

and the Fen district and neighbouring counties, and by contrast the light incidence in Yorkshire, Lincolnshire, Herefordshire and Pembrokeshire. Gradations of correspondence on both maps are evident in comparing Norfolk, Suffolk and Essex ; Kent and Sussex ; Cornwall and Devon ; Glamorgan and Carmarthen.

(3) *Sweden.*

A higher death rate from cancer was shown to exist in two Swedish counties, Kopparberg and Gefleborg, than was found in the total rural areas of Sweden (Stocks, 1925). Those two counties, as shown by examination of schoolchildren, recruits for the army and candidates for confirmation, were found to have a higher incidence of goitre as compared with the rest of the country.

(4) *Norway.*

Similarly, known goitrous counties in Norway were compared by Stocks (1924) with counties showing little goitre, and it was found that the goitrous counties showed a cancer rate for all organs of 113·7 as compared with 94·4 for the non-goitrous counties.

(5) *Iceland.*

In Iceland, the thyroid has been shown to contain one of the highest concentrations of iodine, and endemic goitre is non-existent (Rundle, 1951). Thyroids there weigh on an average 14 g. in men and 11·5 g. in women, as compared with the average of 25 g. in most countries (Kelly, 1946). Now Icelanders are mostly of Norwegian stock with some Danish admixture, and cancer incidence in Norway is 15·32 and in Denmark 16·41, but in Iceland it is only 5·98 (not standardised) (W.H.O., 1952*b*).

(6) *Spain and Portugal.*

Goitre is only mentioned as occurring in one province in Portugal, Alemtejo, on the southern end of the Spanish border (Kelly, 1946). Goitre is common in Spain where there has been a goitre commission for many years. The cancer incidence in Spain is 165 per cent of that found in Portugal.

(7) *Switzerland.*

Switzerland has always had a notorious goitre problem, but in spite of the absence of heavy industry and large towns, and in spite of its reputation for healthy living conditions, this country has always had a high incidence of malignant disease. Stocks (1924) found a close statistical relationship between the goitre rate of the various cantons as calculated from figures taken from the examination of recruits for the army, and carcinoma of the stomach and oesophagus of the general population. When considered canton by canton, the regression figure is $0{\cdot}6459 \pm 0{\cdot}0302$, or if the canton of Ticino is omitted the figure rises to $0{\cdot}7481 \pm 0{\cdot}0554$, which lifts the correlation well beyond the bounds of mere chance. Taking into consideration the lightly affected regions as well as the heavily affected ones it is possible to make out a correlation by inspection, though by no means so clearly as for England and Wales (Fig. 4, 5).

Fig. 4.—Goitre incidence in Switzerland (after Stocks, 1924).

FIG. 5.—Cancer incidence in Switzerland (after Stocks, 1924).

(8) *Germany.*

The crude death rate for carcinoma of the lung among the miners of Schneeberg and Joachimsthal (where the radium for Madame Curie was first mined) is 25 times—not per cent—that of the general rate for carcinoma of the lung for the rest of Germany (Machle and Gregorius, 1948). These mines are fairly close together in one of the four main goitre centres of Germany on the Erzgebirge frontier of Saxony and Czechoslovakia. In these mines all the miners are presumably equally at risk from the inhaled radio-active material, but by no means all the men develop cancer of the lung and it is at least open to speculation that the apparent immunity of a considerable section of the mining community may be due to some factor associated with the presence or absence of goitre, or alternatively to a properly or feebly functioning thyroid. In this connection it is perhaps worth noting that a high incidence of lung cancer has not, so far as I am aware, been reported from other radio-active mines.

(9) *United States of America.*

The large goitre belt in the U.S.A. across most of the northern states and running south along much of the Pacific Coast (Fig. 6), first came to the public

FIG. 6.—Goiter incidence of U.S.A. (after McClendon, 1939).

notice during the Great War when, of nearly 12,000 men turned down for military service on account of goitre, some 3,600 were unable to button the military tunic around their necks on account of the size of their goitres (McClendon, 1939). If the forty large towns and cities of the U.S.A. are arranged in descending order of their crude death rates for cancer as given by Hoffman (1915) it will be found that eight of the first ten lie in known goitrous areas, and that eight of the last ten (showing the lowest cancer rates) lie in the non-goitrous areas. Stocks (1925) has showed that there is a statistical correlation, with a regression figure of $0{\cdot}644 \pm 0{\cdot}065$, between the incidence of goitre, as recorded by the recruit figures, and carcinoma of the stomach and oesophagus, when considering separate states.

(10) *Australia and New Zealand.*

Australia is considerably more industrialised than New Zealand and has many large cities as compared with the latter country. Furthermore, rodent ulcer is infinitely more common in Australia, where goitre does not obtrude as a major health problem, though there are certain areas where there are definite, if small, districts where goitre is found (Kelly, 1946). The Australian cancer death rate is 12·88. The more rural and agricultural population of New Zealand has a death rate from cancer of 14·56. Here goitre is a major health problem and its incidence in Europeans, Maoris and animals alike has been closely studied for years. The Maoris are said to have a word, " tenga ", which has been in their language for centuries and means goitre (Kelly, 1946).

Fig. 7.—World goitre map (after Kelly, 1946).

(11) *Japan.*

In Japan much seaweed and fish is eaten, and goitre is rarely found except for a few cases in the central mountain areas (McClendon, 1939). The islands are densely populated, Tokio and Osaka having greater populations than any town in the United Kingdom except London, and there is much heavy industry and generally a low standard of living. These are conditions usually associated with a high cancer incidence, but for long Japan has enjoyed one of the lowest cancer rates of the world, 4·66 in 1947.

This geographical survey lends support to the thesis not only that the general incidence of malignant disease is to a large extent conditioned by local factors, but that the one factor in evidence is apparently linked with the availability of iodine, using goitre as a clinical indicator.

### CLINICAL OBSERVATIONS.

A self evident association between cancer and goitre or impaired thyroid function from the clinical aspect is unlikely to have remained undetected till now. Although the substance of this enquiry took origin from certain clinical observations, data as clear as the geographical and experimental evidence cannot be expected for some years' time, when the results of the clinical trials and investigations that have been started become available. There are, however, a number of observations that, while not fully conclusive, may provide pointers sufficiently circumstantial to warrant further clinical investigation on the lines outlined here.

(1) The metabolic rate in man decreases progressively with increasing age, and the incidence of malignant disease increases progressively with age. The incidence of malignnat disease thus seems to be correlated with the decrease in metabolic rate. As metabolic rate is in general a rough indication of thyroid activity, there would be appear to be some relationship between malignant disease and thyroid dysfunction. Thus, the lowered metabolic rate often seen in diabetes mellitus (Joslin, 1946) may be considered with the increased liability of diabetics to develop carcinoma in any site or organ as compared with non-diabetics (Joslin, 1946). In the same way, the lowered metabolic rate commonly found in patients with all types of peptic ulcers (Kolmer, 1949) may be considered with the incidence of carcinoma of the stomach—still one of the commonest neoplasms of man.

(2) It has long been observed that puberty and pregnancy are periods when goitres may appear (Marine and Lenhart, 1909 ; Marine, 1935), and may disappear when the period of excessive demand ceases or when iodine is given. These are also the periods when melanomata tend to increase in size and may, not infrequently, become malignant. At the beginning of this year Mrs. R— was admitted to this hospital on account of malignant melanoma with spread to glands in the course of her third pregnancy. In her two previous pregnancies she also developed malignant melanoma on each occasion and was in this hospital in consequence. She has a moderate enlargement of the thyroid of the smooth " colloid " type seen commonly in pregnancy. The end of pregnancy is also the period when chorionic carcinoma and lactation carcinoma of the breast occur—perhaps the most rapidly growing class of cancer known.

(3) Administration of thyroid has been found to improve the action of oestrogens in the treatment of carcinoma of the prostate, permitting the effective use of smaller doses of oestrogen and delaying the onset of insensibility to oestrogen (Winsbury White, 1948). In support of this clinical experience a paper in endocrinological research is quoted (Chu and You, 1945), but the final conclusions of these workers was that the simultaneous administration of oestrogen and thyroid was the same as that obtained by thyroid feeding alone.

(4) Reduction of keloid formation can often be brought about by administration of thyroid. A series of such cases is given by Updegraff (1933), all in his series showing a low metabolic rate. Although keloids are not neoplasms in the strict sense of the word, they tend to recur after adequate excision, and partly ulcerated keloids are prone to undergo carcinomatous change after a period of time.

(5) Weights of the thyroid of children coming to autopsy between the ages of 6 and 10 were observed in three European towns, and the cancer incidence of those noted (Bayard, 1919).

| | Bern. | Munich. | Kiel. |
|---|---|---|---|
| Average thyroid weights | 18·5 g. | 11·9 g. | 7·4 g. |
| Cancer mortality per 100,000 population | 132 | 98 | 57 |

(6) The iodine content of the thyroid has been shown to reach its maximum in the adult and remains fairly contant until the age of about 50, after which there is a gradual decline (Rundle, 1951). The age for the start of the decline in iodine content of the thyroid coincides in general with the age at which malignant disease in man becomes a prominent feature.

(7) In the second week of January of this year there were at least three patients in this hospital at the same time with gross goitre and cancer.

Mrs. M—, carcinoma of breast and a large colloid goitre visible seven or eight beds away down the ward.

Mr. E—, lymphosarcoma and a thyroid that weighed 80 g. at autopsy excluding involvement with growth.

Mr. R—, carcinoma of parotid. The nodular goitre caused some difficulty during resection of glands of neck.

Since January other patients have been seen in the wards of the hospital with coexistent carcinoma and goitre. It is relevant to note that the area from which this hospital draws its material, Gloucester, Somerset and Cornwall, is not one in which goitre is frequent.

(8) The association of goitre and malignant disease in the post-mortem room was strikingly illustrated by analysis of 1000 post mortems at the Middlesex Hospital (Stocks, 1924), when anomalies of the thyroid were found in 13·3 per cent of males and 21·2 per cent of females of 500 persons dying of cancer ; whereas only 2·0 per cent of males and 6·5 per cent of females dying of non-cancerous conditions showed anomalies of the thyroid. Professor Barlow excluded from these anomalies secondary invasion of the thyroid by cancer, and the anomalies most frequently found were simple parenchymatous enlargement and adenomatosis, while calcified nodules, cystic changes, atrophy and fibrosis were frequent. The final result of the survey showed that thyroid anomalies occurred in 18·7 per cent of 500 persons dying of cancer and only in 3·9 per cent of 500 persons dying of conditions other than cancer. Although Stocks (1954, personal communication) has recently cast some doubt on the accuracy of the non-cancerous figures while confirming the cancerous ones, it would require a considerable error of observation to influence greatly the 480 per cent preponderance of thyroid anomalies in the cancerous figures.

The majority of hypo-functioning thyroid glands are small and virtually impossible to detect on palpation and indistinguishable clinically from normally functioning glands, and it is common clinical experience that moderately enlarged thyroids are easily missed even when they are not retrosternal, especially when no symptoms direct attention to that area of the neck. Measurement of radio-iodine clearance and of protein bound iodine are therefore essential steps to establish dysfunction, and these are now being carried out on a series of patients with known malignant disease, some of whom have shown unexpectedly low clearance and P.B.I. values under the age of 50 without other clinical evidence of hypothyroidism. A further series of post-operative cases of carcinoma of various sites, treated with thyroid and other organic iodine preparations is being observed in the follow-up clinics of this hospital, and in due course it should it be possible to report on the results of these investigations.

### ANIMAL EXPERIMENTS.

The influence of thyroid activity on malignant growths in animals is demonstrated in the review of experimental work quoted here, in which both administration and deprivation have been used to show its important effect on dependent neoplasms.

(1) The incidence of successful takes of grafts of granulosa cell tumours, luteomas and tubular adenomas made into the spleen of mice was noted under varying conditions.

| | Successful "takes." | Per cent. |
|---|---|---|
| On normal diet . . . . | 16 out of 21 . . . . . | 76 |
| Same diet plus 0·2 per cent thyroxine (Miller and Gardner, 1950) | 2 out of 20 . . . . . | 10 |

*Comment.*—The presence of excess of thyroxine in the tissues appears to be prejudicial to the successful grafting of tumours from one mouse to another.

(2) The incidence of tumours at the site of injection of chemical carcinogens is reduced by a single injection of thyroxine, and metabolic studies have shown that the rate of disappearance of the carcinogen from mice is significantly increased by thyroxine treatment. Four weeks after injection of 1, 2, 5, 6-dibenzanthracene into mice treated or untreated with thyroxine, the amount of carcinogen remaining in the carcases, estimated spectroscopically, is significantly lower with thyroxine treated animals. Conversely, dibenzanthracene exerts an inhibitory effect on the toxic effect of thyroxine with anoxia survival tissue as the criterion. When the dose response curve of mice to the sarcomagenic action of dibenzanthracene is studied (injections of 5, 16, 48 and 1000 $\mu$g. of DBA) it is found that simultaneous administration of thyroxine significantly lowers the tumour response. This also is true if thyroxine is given at different sites in the animal. Thiouracil feeding markedly increases the incidence of DBA tumours produced by 1 mg. of DBA (Bather, 1952).

(3) Rats bearing Walker Rat Carcinoma 256 were treated with natural thyroxine. Of these 27 per cent showed complete remission and 12 per cent "favourable" histological response, 68 per cent. showing no response at all. Another group treated with synthetic hormone gave only 2 per cent of remissions and no response in 98 per cent of animals. 158 animals were involved (Herbut, Kraemer, and Jacksen, 1950).

*Comment.*—Regression of rat tumours may be induced by thyroxine the natural hormone being superior to the synthetic.

(4) Feeding butter yellow (*p*-dimethylaminobenzene) to rats produced malignant neoplasms of the liver. The addition of wheat, yeast or casein to the diet all delayed the onset of tumours as compared with the controls. (Rusch and Baumann, 1945).

*Comment.*—Wheat, yeast and casein are all proteins with moderately high content of tyrosine, thyroxine being derived by stages from tyrosine and iodine, tyrosine deficiency being a recognised cause of hypothyroidism.

(5) A rat thyroid tumour occurred after prolonged methyl thiouracil administration. This tumour could be transplanted to another rat provided the second rat was first rendered thyroid deficient. In the second " take " rats it underwent anaplastic changes and could then be transplanted into yet other rats without thyroid deficiency. At this stage the tumour had presumably become autonomous after being dependent. (Purves, Griesbach and Kennedy, 1951).

(6) The course of growth of spontaneously occurring mammary tumours in mice was observed. Most had multiple tumours. Many grew conspicuously during pregnancy and regressed after parturition, but recurred promptly when the mouse became pregnant again. Foulds is convinced that the phenomenon is hormonal, but the mechanism remained obscure for apparently it is not due to the action of progesterone or oestrogen. (Foulds, 1949).

*Comment.*—Thyroid deficiency during pregnancy has long been recognised (Marine and Lenhart, 1909 ; Marine, 1935) and might well account for the findings of this experiment.

This review of a selection of experimental work designed to show the action of thyroid in tumour production and regression, contributes further support to the geographical and clinical observations already set out to correlate thyroid deficiency and cancer incidence.

### PHYSIOLOGICAL AND PATHOLOGICAL DATA.

Under normal conditions the thyroid is closely associated with (*a*) Tissue Oxidation, (*b*) Growth, (*c*) Development. These three functions are conveniently compared with conditions that exist in malignant neoplasms.

*Tissue oxidation.*

Thyroid stimulates metabolism by increasing oxygen consumption, probably by catalysing the enzyme systems which are responsible for tissue oxidation processes.

Malignant tissues, in comparison with normal tissues and benign tumours, are characterised not only by having the lowest concentration of Cytochrome C, but also by having the greatest disparity between the components of the oxidase–cytochrome system. Malignant tissues also show very low amounts of catalase and of the flavin enzymes and co-enzymes (Greenstein, 1947).

*Growth.*

Thyroid promotes normal growth under normal physiological conditions.

Most carcinogenic substances, in particular the potent hydrocarbons but also those of the urethane group, have been shown to have a considerable growth restraining influence (Haddow, 1938).

*Development.*

Thyroid brings about development and differentiation of tissue.

Histologically, malignant disease tends to show a dedifferentiation or reversion towards anaplastic tissue pattern that is often compared to embryonic or foetal type of cell.

### DISCUSSION.

It is instructive to put these observations on a footing with the present knowledge of tumour regression.

First, it may be recalled that it has been variously estimated that spontaneous regression of tumours occurs in man once in about 5000 cases of malignant disease.

This in itself commends to many the idea that cancer has a chemical and probably a hormonal basis, for comparatively sudden changes in the function of endocrine glands either to the plus or to the minus direction is a familiar observation, and in particular with regard to the thyroid.

Secondly, it is relevant to note the comparative frequency of multiple primary malignant neoplasms, for this interesting condition suggests that the body can fairly readily develop a malignant diathesis, and the most likely explanation for this is a hormonal change, the sites of the primary growths being determined by siting factors, which are better understood but which are not within the scope of this present enquiry. A survey by Williamson (1950) of the work of well known English and Continental morbid anatomists gives a figure of 4 per cent for the incidence of multiple primary carcinomata of all autopsies of malignant cases. In view of the comparatively short expectation of life in the average patient with cancer this figure of 4 per cent is probably higher than might at first appear, for the time available to these persons to develop fresh primaries is inevitably short. Furthermore, when it is remembered how easy it is to regard every mass of growth both at the bedside and at autopsy, as an " obvious " metastasis, it is perhaps not altogether surprising that this figure is not higher. Nevertheless, multiple primary neoplasms, even apart from mesenchymal tumours, are common experience to both clinician and morbid anatomist alike, and some would go so far as to think that their numbers are on the increase, or possibly that they are being recognised more freely.

TABLE II.—*Incidence of Multiple Primary Carcinoma.*
(After Williamson, 1950.)
Synchronous double primary carcinomata.

| Author. | Number of necropsies. | Number of malignant cases. | Number of multiple malignancy. |
|---|---|---|---|
| Hanlon | 3000 | 950 | 18 (1·9%) |
| Bilello | 8024 | 1154 | 7 (0·5%) |
| Bugher | 4394 | 983 | 30 (3·1%) |
| Austin | 8124 | 887 | 24 (2·7%) |
| Burke | 2033 | 583 | 46 (7·8%) |
| Tullis | 6836 | 1044 | 21 (2·0%) |
| Warren and Gates | — | 1075 | 40 (3·7%) |
| Warren and Ehrenreich | — | 2829 | 194 (6·8%) |
| Total | — | 9495 | 380 |

Average rate of multiple primary malignancy = 4·0 per cent.

Unfortunately it is not possible in the present state of knowledge to give any accurate account of the effect of hormones on the observed temporary regression of tumours either in man or in animals, for in this field there are too many gaps in our knowledge, too many uncertainties and a certain residuum of conflicting evidence. But the results of adrenalectomy, castration and the administration of oestrogens and sometimes of androgens, all seem to point to a suppression, if only temporary, of some of the functions of the pituitary, at least in man. The recent work of Moon, Simpson, Li and Evans (1950*a*, 1950*b*, 1952*a*, 1952*b*) has convincingly confirmed the conclusions of earlier workers twenty years ago (Ball and Samuels, 1932 ; Bischoff, Maxwell and Ullmann, 1934), that whereas extracts of the anterior pituitary cause tumours to arise in a variety of sites in laboratory rodents, extirpation or irradiation of the pituitary will either slow up the rate of established tumour growth or will effectively inhibit the carcinogenic effect of such powerful substances as methylcholanthrene. Recently hypophysectomy by Luft and Olivecrona (1953) in a series of women with advanced mammary carcinoma who had already been treated with every standard surgical hormonal, and radiation therapy without effect, appears to have reproduced in man the results observed in animals twenty years ago.

Attempts to bring about tumour regression by castration or adrenalectomy are, for the most part, merely applications of those animal experiments, but acting through their indirect effect on the pituitary. The administration of oestrogens aims at bringing about a partial, if temporary, medical hypophysectomy, though unfortunately oestrogens and androgens have been shown to possess moderately active carcinogenic properties (though not the synthetic stilboestrol), and that oestrogens have in addition a noticeable mitogenic effect on sensitive tissues (Bullough, 1942 1946, 1950*a*, 1950*b*). The final drawback to oestrogen therapy, apart from its minor side effects, is that the pituitary, after a period of medication, becomes indifferent to the action of stilboestrol. Indeed, it was shown by Burroughs and Horning (1947) that if the pituitary is given a prolonged treatment with oestrogens the anterior lobe as a whole passes into a stage of hyperplasia having all the characters of an adenoma.

In common with other endocrine glands, the thyroid has a reciprocal relationship with the pituitary. Thus, the gonadotrophic hormone from the pituitary will stimulate the ovary to produce oestrogen and the blood level of oestrogen so produced in turn governs the subsequent output of gonadotrophic hormone, a low oestrogen level leading to a high output of gonadotrophic hormone and *vice-versa*, a high oestrogen blood level leading to a low gonadotrophic hormone output by the pituitary. In the same way, a low thyroxine level in the blood will act as a stimulus to the pituitary which will put out increased amount of thyrotropic hormone to stimulate, in its turn, the thyroid ; and conversely, a high thyroxine level in the blood has the effect of suppressing the activity of the pituitary. Thus both oestrogen and thyroxine have a similar action on the pituitary and tend to suppress its activity, but in addition thyroxine has not been shown to possess any carcinogenic properties, nor would such activity be expected from consideration of its molecular structure ; nor has it been shown that the pituitary eventually becomes indifferent to its action. Furthermore, the presence of thyroxine in the tissues, as has already been amply demonstrated in the animal experiments detailed above, brings about a tissue environment that is unfavourable to tumour growth and development, at least as long as tumours remain in the dependent phase.

In an attempt to explain how this change in tissue is effective we are left with several possibilities :

(*a*) That thyroxine encourages normal physiological tissue respiration rather than the so-called anaerobic type which appears to be the one demonstrable biochemical difference between normal and neoplastic tissue (Greenstein, 1947).

(*b*) That thyroxine in adequate amounts may reverse the process of dedifferentiation or drift towards anaplastic growth that characterises most neoplastic tissue—the more malignant, the more anaplastic—that is, it may bring about the process of differentiation and development that it certainly does promote in the embryonic stages and early years of life.

(*c*) That thyroxine, by raising the metabolic rate or by maintaining it at a proper level, brings about some degree of katabolism and/or an increased rate of excretion of the carcinogen.

(*d*) That thyroxine brings about some depression of pituitary activity. In this connection, the depolymerisation of connective tissue which seems to be brought about by excess of thyrotropic hormone (Robb-Smith, 1954) is particularly relevant in view of the importance which some attach to changes in connective tissue in the genesis of malignant disease, for thyroid medication is capable of reversing the process and of bringing about polymerisation of the mucopolysaccharides and so a return to a more normal structure of connective tissue.

(*e*) That the favourable effect of thyroxine in these experiments is due to a combination of some or all of these factors.

At a recent Imperial Cancer Research Fund lecture at the Royal College of Surgeons, Professor Hadfield said : " It is my firm belief that for many years we have been so anxious to discover all we possibly can about the structure of the growth and its metastases that we have almost forgotten the soil in which it grows." (Hadfield, 1954.)

In coming to any conclusion regarding the possible association between malignant disease and thyroid function, it cannot be too strongly stressed that a low metabolic rate or an insufficiency of thyroid substance can in no way be considered as a primary cause of cancer. Thyroid and related substances cannot therefore be considered as a cure for cancer. It is, however, suggested that thyroid function (or dysfunction) may be associated with the susceptibility or immunity to cancer. As such, thyroid might well be used as a therapeutic weapon, ancillary only to accepted surgical treatment, much as antiserum is a valuable ancillary in the treatment of tetanus or gas gangrene, but in itself is no substitute for radical surgery in either condition, even though it may rightly be regarded as the factor which takes suitable surgical toilet out of the realm of debatable value to the sphere of accepted success under reasonable conditions.

In the wider and perhaps more important field of preventive medicine, the possibility that an increased susceptibility to cancer occurs in those with a poor thyroid function leads for the first time to a real chance of adopting prophylactic measures against cancer on a wide scale. The measures adopted would be in the main those already available against goitre in the young and in adolescents, with the idea of building up healthy active thyroid glands during their period of development ; and in adult life steps would be taken to maintain a good level of thyroid activity by ensuring that iodine is available in suitable quantities in food and drink, especially when middle life is reached and there is a natural tendency for the iodine level to fall (Rundle, 1951). Only in some such way can an immunity be built up against the many carcinogenic substances which it is virtually impossible to avoid, largely due to the widespread use of coal and oil. In addition, it must be recalled that there are substances such as cholesterol, vitamin D and the sex hormones and even sunshine itself, which are all part of a normal and healthy existence in spite of their proved capacities to cause cancer, even if they are not finally shown to be the primary causes of the commonly occurring, non-industrial neoplasms that form the bulk of the problem of malignant disease.

### SUMMARY.

Data from fifteen countries in four continents give support to the importance of local factors to account for the known local variations of cancer incidence.

Iodine availability, traced by goitre incidence, appears to be one of such factors. Closer scrutiny of some of these countries corroborates these conclusions.

Clinical findings and a review of some of the experimental work available lend further emphasis to these observations.

The apparent relationship of thyroid insufficiency and the liability to develop cancer is discussed in connection with such other hormonal influences over cancer as are already known, particularly in respect of the pituitary.

Lines of investigation and clinical trials have been started, but results of any value cannot be expected for several years.

A non-specific organ immunity or susceptibility seems to be the simplest explanation of the facts presented, and the possibilities, preventive and perhaps therapeutic, that are opened up by this line of research are briefly discussed.

I am indebted to Dr. Polak of Amsterdam for Fig. 1 and for much information regarding Holland ; to Professor McClendon and the Minnesota Press for Fig. 6 ; to Dr. Stocks for Fig. 2, 4, and 5 and for advice and encouragement ; to the B.E.C.C. for permission to publish Fig. 2 ; to Mr. McEwan for much helpful criticism and for Fig. 3 ; to Dr. Williamson for Fig. 8 ; to Dr. Kelly for Fig. 7 and for much factual material ; and to Mr. Banham for the reproductions of the maps.

I am especially grateful to Dr. G. Price of the Bristol Cancer Research Institute for much kindly advice and, finally, to colleagues at Frenchay and Bristol General Hospitals for access to many cases under their care.

### REFERENCES.

BALL, H. A., AND SAMUELS, D. T.—(1932) *Amer. J. Cancer*, **16**, 351.
BATHER, R.—(1952) *Cancer Res.*, **12**, 247.
BAYARD, O.—(1919) ' Beitrage zur Schilddrusenfrage ', Basel (Schwabe), p. 42.
BISHOFF, F., MAXWELL, L. C., AND ULLMANN, H. J.—(1934) *Amer. J. Cancer*, **21**, 329.
BULLOUGH, W. S.—(1942) *J. Endocrin.*, **3**, 150.—(1946) *Philos. Trans.*, B, **231**, 453.—(1950*a*) *J. Endocrin.* **6**, 340.—(1950*b*) *Ibid.*, **6**, 350.
BURROWS, H., AND HORNING, E. S.—(1947) *Brit. med. Bull.* **4**, 367.
CAMPBELL, J. M. H.—(1925) *Quart. J. Med.*, **18**, 191.—(1927) *J. Hyg., Camb.*, **26**, 1.
CHU, J. P., AND YOU, S. S.—(1945) *J. Endocrin.* **4**, 115.
CLINQUART, E.—(1926) *Bull. Acad. Méd. Belg.*, **6**, 505.
FOULDS, L.—(1949) *Brit. J. Cancer*, **3**, 345.
GREENSTEIN, P.—(1947) ' Biochemistry of Cancer '. New York (Academic Press).
HADDOW, A. J.—(1938) *J. Path. Bact.*, **47**, 567.
HADFIELD, G.—(1954) *Ann. Roy. Coll. Surg.*, **14**, 21.
HERBUT, P. A., KRAEMER, W. H. AND JACKSEN, J.—(1950) *Cancer Res.*, **10**, 224.
HOFFMAN, F. L.—(1915) ' The Mortality from Cancer throughout the World.' Newark (Prudential Press), pp. 122, 128.
JOSLIN, E. P.—(1946) ' The Treatment of Diabetes mellitus,' 8th ed. London (Kimpton), pp. 262, 653.
KELLY, F. C.—(1946) ' World Goitre Survey.' London (Iodine Educational Bureau).
KOLMER, J. A.—(1949) Laboratory Examinations, 2nd ed. New York (Appleton Century), p. 177.
LEGON, C. D.—(1951) *Brit. J. Cancer*, **5**, 175.—(1952) *Brit. med. J.*, ii, 700.
LUFT, R., AND OLIVECRONA, H.—(1953) *J. Neurosurg.* **10**, 301.
MACHLE, W., AND GREGORIUS, F. (1948) *Congr. int. Mal. prof.*, **9**, 463.

MARINE, D.—(1935) *J. Amer. med. Ass.*, **104**, 2334.
*Idem* AND LENHART, C. H.—(1909) *Arch. intern. Med.*, **4**, 440.
MCCLENDON, J. F.—(1939) 'Iodine and the Incidence of Goiter.' Minneapolis (University of Minnesota Press.)
MCEWAN, P.—(1938) *Brit. med. J.*, i, 1037.—(1948) 'The Clinical Picture of Thyrotoxicosis.' Edinburgh (Oliver & Boyd).
MILLER, O. J., AND GARDNER, W. U.—(1950) *Cancer Res.*, **10**, 233.
MOON, H. D., SIMPSON, M. E., LI, C. H., AND EVANS, H. M.—(1950*a*) *Cancer Res.*, **10**, 297.—(1950*b*) *Ibid.*, **10**, 549.—(1952*a*) *Ibid.*, **12**, 448.—(1952*b*) *Science*, **116**, 331.
MURRAY, M. M., RYLE, J. A., SIMPSON, B. W., AND WILSON, D. C.—(1948) M.R.C., Memorandum 18. London (H.M.S.O.).
NORDLING, C. O.—(1953) *Brit. J. Cancer*, **7**, 68.
PURVES, H. D., GRIESBACH, W. E., AND KENNEDY, T. H.—(1951) *Brit. J. Cancer*, **5**, 301.
ROBB-SMITH, A. H. L.—(1954) 'Lectures on General Pathology,' p. 567. London. (Lloyd-Luke).
RUNDLE, F. F.—(1951) 'Joll's Diseases of the Thyroid.' London (Heinemann), p. 78.
RUSCH, H. P., AND BAUMANN, C. A.—(1945) Research Conference on Cancer, Washington.
SOURASKY, M.—(1928) 'International London Conference on Cancer.' Bristol (John Wright), p. 536.
STOCKS, P.—(1924) *Biometrika*, **16**, 364.—(1925) *Ibid.*, **17**, 159.—(1928) 'International London Conference on Cancer.' Bristol (John Wright), p. 508.—(1947) 'Studies on Medical Population Subjects.' No. 1. London (H.M.S.O.).—(1950) *Brit. J. Cancer*, **4**, 147.—(1953) *Brit. med. J.*, ii, 847.
UPDEGRAFF, H. L.—(1933) *J. Amer. med. Ass.*, **101**, 1138.
WAYNE, E. J.—(1954) *Brit. med. J.*, i, 411.
WILLIAMSON, T. B.—(1950) *Ibid.*, i, 648.
WINSBURY-WHITE, H. P.—(1948) 'Textbook of Genito-Urinary Surgery.' Edinburgh (Livingstone), p. 525.
World Health Organisation. (1952*a*) *Epidem. vit. Stat. Rep.*, **5**, 1.—(1952*b*) *Ibid.*, **5**, 371.